# Medicaid Becomes the First Third-Party Payer to Cover Passive Remote Monitoring for Home Care: Policy Analysis

**DOI:** 10.2196/jmir.9650

**Published:** 2018-02-21

**Authors:** Clara Berridge

**Affiliations:** ^1^ School of Social Work University of Washington Seattle, WA United States

**Keywords:** policy making, Medicaid, long-term care, aging, information technology, passive remote monitoring, sensing, ethics, technology implementation

## Abstract

**Background:**

Recent years have seen an influx of location-tracking, activity-monitoring sensors, and Web-cameras to remotely monitor the safety of older adults in their homes and to reduce reliance on in-person assistance. The state of research on these monitoring technologies leaves open crucial financial, social, and ethical cost-benefit questions, which have prevented widespread use. Medicaid is now the first large third-party payer in the United States to pay for these technologies, and their use is likely to increase as states transition to managed long-term services and supports (MLTSS).

**Objectives:**

This is the first study to examine how state Medicaid programs are treating passive remote monitoring technologies. This study identifies (1) which states allow location tracking, sensor systems, and cameras; (2) what policies are in place to track their use; (3) what implementation processes and program monitoring mechanisms are in place; and (4) what related insights Medicaid program stakeholders would like to learn from researchers.

**Methods:**

Interviews were conducted with 43 state, federal, and managed care organization (MCO) Medicaid program stakeholders about how these technologies are used in state waivers serving community-dwelling older adults in 15 states, and what policies are in place to regulate them. The interviews were analyzed by the research team using the framework analysis method for applied policy research.

**Results:**

Two-thirds of the states cover location tracking and activity-monitoring sensors and one-third cover cameras, but only 3 states have specific service categories that allow them to track when they are paying for any of these technologies, impeding regulation and understanding of their use at the state and federal level. Consideration of ethical and social risks is limited, and states struggle to understand which circumstances warrant use. They are further challenged by extreme resource restrictions and transitions to MLTSS by MCOs inexperienced in serving this growing “high-need, high-cost” population.

**Conclusions:**

Decisions about Medicaid reimbursement of technologies that have the potential to dramatically alter the way older adults receive supportive services are being made without research on their use, social and ethical implications, or outcomes. At a minimum, new service categories are needed to enable oversight. Participants prioritized 3 research aims to inform practice: (1) determine cost-effectiveness; (2) identify what type of information beneficiaries want to be generated and whom they want it to be shared with; and (3) understand how to support ethical decision making for beneficiaries with cognitive impairment. These findings provide direction for future research and reveal that greater interaction between policy makers and researchers in this field is needed.

## Introduction

Most are aware of the statistic that by 2050, 20% of the US population will be aged 65 years or older [1]. The silver tsunami, age wave, and other catastrophic weather metaphors are commonly evoked along with the “care crisis” due to the changing ratio of elder care supply and demand. Among the 43+ million Americans who are over the age of 64 years today, nearly 1 in 3 live alone and half will experience severe cognitive impairment or need long-term care for at least 2 activities of daily living at some point over the remainder of their lives [2,3]. At a national average of US $20 per hour, having a regular home health aide to provide this care is costly [4], but it amounts to roughly half the cost of the median shared nursing home room at about $86,000 per year [4]. Enabling aging-in-place is widely considered a priority in the context of a shortage of human and financial resources for elder care [5-7]. The population of older adults living in nursing homes has declined significantly over the past few decades [8], and despite the rise of assisted living facilities, 80% of older adults who receive assistance live in private homes in the community [1].

While the majority of the population receives ongoing assistance in their own homes, this is not a covered service under Medicare. Medicaid pays for the largest share of long-term services and supports (LTSS), which includes both residential and home and community-based care. Two Medicaid LTSS trends are noteworthy. First, there has been an increase in the portion of Medicaid LTSS expenditures on home and community-based services (HCBS) compared with nursing homes, reflecting the goals of rebalancing initiatives. In 2015, 44% of LTSS expenditures on programs for older adults and people with physical disabilities were accounted for by HCBS [9], a portion that has more than doubled since 1995 [10]. A second trend is the expansion over a short period of time of managed long-term services and supports (MLTSS) where managed care organizations (MCOs) contract with states to provide services to Medicaid beneficiaries. As of February 2017, 19 states had transitioned to MLTSS [11].

State Medicaid waivers are a key component of many Centers for Medicaid and Medicare Services (CMS) and state rebalancing initiatives to reduce reliance on institutional care, with the 1915(c) waiver known as the “home and community-based services waiver” accounting for more than half of all HCBS Medicaid spending [9]. These waivers, whether contracted through providers or MCOs in MLTSS states, are states’ most flexible mechanism to connect people with services such as case management, home health aides, personal care, and respite care, to prevent or delay nursing facility admissions or to move people out of institutions. Waivers are a source of variation in programs across states because they allow states to waive federal regulations to, for example, target services to specific populations such as those at risk for institutionalization and to demonstrate new methods for providing services [12].

### The Rise of Passive Remote Monitoring Technology in Elder Care

Recent years have seen an influx of accessible, low-cost technologies for continuous passive remote monitoring in the form of Web-cameras and sensor systems that monitor activity and movement in and out of the home [13]. Passive remote monitoring systems collect and transmit a range of data, including location outside the home (GPS), movement and activity (sensor systems), and camera recordings of activity in intimate living spaces. With these technological developments, providers of Medicaid-funded LTSS are turning to passive remote monitoring technologies to enable what is referred to as “most integrated housing” and to reduce costs of services for waiver beneficiaries.

In the United States, barriers to widespread uptake of passive monitoring technology have been the lack of reimbursement by third-party payers and inadequate evidence of clinical and financial benefit [14,15]. Medicaid is the first large third-party payer to begin to formally reimburse these technologies for the care of older adults, but this trend has not been cited in academic literature, and virtually nothing is known about it. There are no national data on the prevalence of their use, nor are there state or federal administrative record capture systems on the use of this category of technology.

The introduction of passive remote monitoring technologies through Medicaid is important because these technologies’ potential for revolutionizing independent living is one of the most widely discussed topics in aging health studies [16]. The past decade has seen a high level of innovation in technology for aging, with numerous governments investing in significant research collaborations, such as the cross-national Ambient Assisted Living Joint Platform of the European Union and the national AGE-WELL Initiative in Canada, launched in 2008 and 2015, respectively. Still, today’s published research is more focused on development rather than the evaluation of impact of devices on health outcomes and lives of older adults [17]. These technologies hold promise to safely supplement and reduce in-person care, but reviews of the English language literature find that they are being deployed with neither evidence of benefit on individual nor systems outcomes [16-22]. Furthermore, while more attention is paid by researchers in Europe and in Canada to ethical challenges, we neither understand how to mitigate the risks that older adults face and which pose significant ethical concerns yet [23-25] nor do we know how to provide comprehensive and effective systems [6] or how to interpret changes detected by monitoring systems in ways that would enable the prediction of adverse events necessary for an intervention [15]. As a result, decisions about Medicaid reimbursement that could dramatically alter the way older adults receive supportive services are being made in the absence of research on the trade-offs in privacy, autonomy, and human interaction, as well as other risks identified by researchers [16]. This is compounded by a lack of clarity about the relevant stakeholders’ perspectives, particularly those who are asked (though this step cannot be assumed) to subject themselves to remote monitoring.

### What Are the Potential Risks and Benefits?

Potential benefits of passive remote monitoring include reduced health service use [26], enhanced emergency response, fall detection, independence and postponement of institutionalization [27], feelings of security and peace of mind [28], whereas potential risks include isolation through reduced human interaction and hands-on care, privacy invasion, loss of control, data inaccuracy [27,29-32], and reduced behavioral autonomy and access to services [29,33,34]. Reviews of the passive remote monitoring literature exhibit that ethical issues are treated superficially, and detailed consideration of serious ethical challenges is absent [23,35]. Authors of a review of ethics in the remote monitoring of those with memory loss conclude that these technologies “pose serious ethical challenges” that “urgently need further analysis” [23]. Gerontologists have also pointed toward the parallels between monitoring and “processes of institutionalization,” whereby deviations from one’s typical behavior or routine lead to the involvement of caregivers [29]. Weber and colleagues warn that individuals will “lack full autonomy in their decision making” when they “normalize” their behaviors while being monitored, thus impeding informational self-determination and the right to be nonconformist [36].

There are few studies of older adults’ attitudes toward these technologies, and many of those that exist are of low methodological quality [37,38]. Small-scale studies of older adults indicate that they want control over decision making about who has access to what data and under what conditions [39,40]. They do not want passive remote monitoring to reduce social interaction, replace human contact, or replace hands-on care [18,39], and they largely reject the collection of visual or audio data recorded by cameras [41,42]. The majority of the research identifies tensions among values such as privacy, independence, and safety, referred to in the literature as “sacrifices” or “trade-offs,” which older adults would make to forestall residential care [24,43,44]. This trade-off framing posits that diminished privacy, autonomy, human interaction, and other risks are outweighed by new efficient means of enhancing safety, reducing hospitalizations, and allowing people to remain living in the community.

These tensions and concerns cited by older adults are largely due to the shift from *active* emergency alarm systems to *passive* monitoring. Unlike personal emergency response systems (PERS) that require the user to push a button, passive monitoring systems collect and transmit data about the type and frequency of activity in a home without the beneficiary having to take any action or even be cognizant of the monitoring. Passive systems are unlike telehealth because there is no communication between patient and provider. Location tracking, activity monitoring sensor systems, and cameras are distinct from the category of remote patient monitoring that references biometrics monitoring, such as heart rate. Sensor-based passive monitoring, for example, uses algorithms to track behavior or movement for interpretation as behavioral biomarkers--in theory, a urinary tract infection might be detected if the activity monitoring system captures a change in an individual’s frequency of bathroom use. Another option is monitoring cameras, which now come in many inexpensive forms purchased by family members, LTSS providers, or facilities wishing to keep an eye on vulnerable older adults. When implementation is advancing faster than the research on what constitutes appropriate and ethical use for different populations, it is important to explore what devices are being used and how.

The aim of this research is to begin casting light on the use and regulation of passive remote monitoring technologies by state Medicaid waiver programs for older adults. The Center for Connected Health Policy publishes state technology coverage based on publically available documents, but this information is incomplete because passive remote monitoring is rarely captured in waiver documentation, which CMS provides on their website. This interview-based policy analysis addressed the following questions in a sample of 15 diverse states:

- Which states allow GPS, sensor systems, and cameras, and under what conditions?
- What policies are in place to track and regulate their use?
- What implementation processes and program monitoring mechanisms are in place?

The urgency of these questions is heightened by both Medicaid HCBS expenditure growth and the possibility of Medicaid becoming a block grant program. These pressures and the absence of research on the circumstances under which passive remote monitoring is a viable and preferable HCBS policy option might propel states and MCOs to substitute it for costly in-person care. Proposals to limit funding growth based on medical inflation are projected to adversely impact the main drivers of state spending, particularly disabled LTSS beneficiaries [45]. Changes in Medicaid payment policy will likely foster greater growth in the use of monitoring technologies, including the prospect of monitoring being an allowable cost under dual eligible Medicaid Advantage Programs. As such, there is a pressing need to learn how these technologies are being used, how their outcomes are monitored, and how their deployment is overseen.

## Methods

This targeted policy analysis included telephone interviews with 43 participants about GPS-based location tracking outside the home, activity monitoring sensor systems, and cameras in aging waivers. To ensure that the most knowledgeable people participated, those who held the following positions were recruited: (1) the manager of one or multiple Medicaid waivers that serve older adults in the community (1915b/c and 1115 waivers), (2) state employees suggested by managers for their institutional knowledge and content expertise, (3) state-level MCO representatives of the largest MLTSS programs referred by state managers, and (4) HCBS policy experts who study participants identified as their “go-to” resource on the use of remote monitoring technologies. In total, 7 participants were content and policy experts at the national level, and 36 participants represented state Medicaid waiver programs or MCOs serving older adults in a diverse sample of states.

The sample of states was selected to capture variation in LTSS policy and include those that are known to be taking the first steps toward expanding services, as well as those that are likely to be the most influential, based on their track records as innovators in HCBS. These 15 states included California, Florida, Illinois, Indiana, Kansas, Massachusetts, Michigan, Minnesota, Missouri, Montana, New York, Pennsylvania, Tennessee, Virginia, and Washington. In this study’s sample, 8 of the 15 states had MLTSS programs, and 4 state branch representatives of large MCOs that provide MLTSS were interviewed about their state’s coverage. The interview protocol is highly targeted because it was informed by pilot interviews with consultants who advise states on these issues, national membership associations, and federal policy makers.

Before each interview, we read each state’s current CMS-approved aging waivers to understand under which service category, if any, that state had been approved to cover any of our 3 categories of technologies (location tracking, sensor systems, and cameras). We cross walked that information into the interviews to clarify discrepancies between what the participants were telling us and the content of the waivers. This also allowed us to confirm that we were speaking about the same specific service categories when participants described their content.

The author and 2 Master of Public Health student researchers analyzed all interview notes and approved waivers using the framework analysis method that was developed for applied policy research [46,47]. This method enables transparent interpretation of specific policies in relation to specific research questions [46]. Framework analysis involved 5 steps: familiarizing, identifying an analytical framework, indexing, charting, and interpreting [46]. We began by thoroughly reading the interview notes and waivers and noting initial analytic observations, followed by classifying the data through an a priori coding scheme based on the questions in the structured interview protocol. The research team met weekly to discuss these codes and applied this framework to a subset of interviews to reach consensus on its application. We then applied the analytical framework to all documents by connecting codes to corresponding portions of the data (indexing). Data from each state were then summarized and arranged into matrices with headings and subheadings developed according to the analytic framework. This visualization drew attention to patterns, trends, and differences between states that guided and clarified our interpretation [47].

## Results

Coverage policies regarding these technologies ranged from explicitly and operationally prohibiting them to explicitly and operationally covering them. As depicted in Table 1, ten of the 15 states cover GPS location tracking systems. Nine states allow sensors, and an additional 3 states report that there is no policy in place regarding sensors. For the allowing states, sensors fall under a broad range of service categories. Sensor-based systems that track location and activity have been integrated into ubiquitous services such as PERS, so that companies now offer multiple functions in familiar products. Decision makers in aging services view these as promising tools. As one state manager explained, “This whole thing is in its infancy right now and we need to incorporate it into our program because it’s one way to save costs, and it’s less invasive and hopefully less costly and more convenient.” MCO representatives echoed these sentiments.

Respondents report that providers of adult family homes originally promoted the use of cameras through the intellectual and developmental disability waivers. In the context of a workforce shortage, providers argued that replacing an aide who periodically checks in at night with a camera in residents’ bedrooms is more efficient and less intrusive. This use of Web-based cameras is still more prevalent in disability waivers than aging waivers. Five of the 15 states cover cameras in at least one of their waivers for older adults. These include a state that covers cameras through an “in lieu of” clause for MCOs, one in the form of baby monitors that are paid for by a waiver, and a third with baby monitors allowable under “Specialized Medical Equipment and Supplies.” Three additional states do not prohibit cameras and reported that they would fall under the “Assistive Technology” service category.

### What Policies Are in Place to Track Their Use?

Service category titles under which passive remote monitoring technologies are covered in practice under the approved waivers are listed in Table 1. Only 3 states collect service data that allow them to track the number of beneficiaries using a particular technology. In Washington, PERS + GPS is assigned an extra “modifier” in administrative records that makes it possible to run a data search to learn the number of people using PERS + GPS tracking. Massachusetts covers GPS under a specific service category called “Home Based Wandering Response Systems,” and “Telecare” is a unique service category in Pennsylvania for sensors. None of the respondents accessed these data for our interviews or at follow-up upon request, noting that it is an onerous process. In all other states, these technologies fall under broader service categories, so the precise type of technology (ie, GPS, sensor, or camera) is unknown to state administrative systems.

State representatives report struggling to collect data that can inform them about what is being used without causing the waivers to be restricted to specific technologies. They felt current service categories were inadequate. “Assistive Technology,” for example, might encompass walkers, screen readers, brail embossers, and a range of other assistive technologies, which are defined as those that maintain or improve functional capabilities. One participant hoped that a specific service category or code for remote monitoring would be developed:

...because we now use the one code for assistive technology, but that doesn’t give us access to the level of detail to understand how that benefit is being used.

Worried about being able to keep up with tech innovation, the participant stated:

I never want what we can do to be limited if a code doesn’t exist for it.

Many wanted to know what other states were doing:

It would be easier if we had a broad category as new technology becomes available, rather than going through a new approval process, but knowing CMS, they want all kinds of specifics. We can’t put just anything in there, of course. Have any states figured it out?

**Table 1:** Summary of state technology coverage, tracking, and policy.

Category	Location tracking	Sensors	Cameras or baby monitors
States prohibiting (15 possible), n	4	3	7
States that do not prohibit but lack policy, n	1	3	3
States covering, whether or not specified in waiver, n	10	9	5
States able to track use, n	2	1	1
States that require special committee, consenting, or rights notification process, n	0	1	1 (if camera is to be used in a bedroom)
Service categories used in practice	PERS; Specialized Medical Equipment and Supplies; Communication *Technology-Specific Categories:* “Home-based Wandering Response Systems” (MA); PERS+GPS modifier under “PERS” (WA)	Goods and Services; Specialized Medical Equipment and Supplies; Assistive Technology; Communication; Possible for MCOs under “Cost Effective Alternative Services” *Technology-Specific Category:* “Telecare” (PA)	Assistive Technology; Specialized Medical Equipment and Supplies; Communication; Environmental Modifications; Possible for MCOs under “In Lieu Of” Clause

Two managed LTSS states reported clauses that allow MCOs to provide services that may fall outside the approved program services, termed “cost-effective alternative services” in one and “the ‘in lieu of’ benefit” in the other. This provides flexibility for MCOs to integrate technologies and other alternatives to standard services outlined in the waivers. States’ oversight of which specific services are provided is minimal. As one waiver manager explained:

What’s hard is that while we get full encounters on their expenditures for “Assistive Technology,” I have no way of knowing the kinds of things they are purchasing for members. I expect that many of these technologies are things they have and would purchase but my encounter data isn’t that granular.

In MLTSS states, MCOs authorize technology and directly contract with providers. States do not know what technology-based services they provide and access care plans only if an issue has been brought to their attention.

The finding that states are largely unable to track what technologies are being covered in practice supports one participant’s characterization:

It’s the wild West.

The inclusion of passive monitoring technologies under broad service categories prevents states or CMS from knowing when providers use passive monitoring.

### What Implementation and Oversight Mechanisms Are in Place?

The risks of these technologies identified in the extant research are not directly addressed by current state Medicaid policies, and there is limited discussion in state aging waiver programs about these risks. With just 1 exception, states that covered any of these technologies lacked consenting processes and monitoring mechanisms. Conditions for use are the same as those that would qualify beneficiaries for the broader service category under which a given technology might fall (eg, PERS), in addition to requirements for an associated behavior, such as wandering behavior when GPS is to be covered. States also report struggling with the weak evidence base and were unclear about which circumstances warranted use, and consequently, trainings were not provided to educate support coordinators who approve services. The additional barriers of a widespread shortage of qualified coordinators and uneven availability of technologies across various regions were often cited.

At the time of these interviews, Minnesota had made the most progress toward formalizing a process for approving cameras and sensors that collect data. Camera requests are reviewed by a committee, and a series of forms recently required by CMS are used to notify beneficiaries and roommates of their rights. Cameras were in use in other waivers where monitoring technology substitutes for staff, but an older adult case had not yet been reviewed. Minnesota was also in the process with CMS of reviewing forms for technology that collect data, including movement sensors, noting that they had always paid for such technology for older adults but had no way of monitoring it or explaining beneficiaries’ rights. Apart from that, however, there are no processes in place to ensure informed consent, discuss the implications of being remotely monitored with beneficiaries, or guided dialogue about privacy or other impacted values. Requests from families, providers, or support coordinators are addressed on a case-by-case basis with no unique process.

The lack of process notwithstanding, the majority of individual respondents acknowledged the potential for ethical problems to arise, though one-third noted that their offices had never discussed or considered potential ethical issues related to these 3 categories of monitoring. Some felt that the inherent risks to privacy of sensors and GPS are outweighed by benefits to safety, offering examples where beneficiaries with dementia had wandered off and gotten lost. In some states where sensor-based systems are permitted, managers noted that restriction of patients’ rights is a concern. Others had questions about efficacy:

I think with the sensing, I guess what’s the most effective. I mean with floor mats or bed mats, do you really just get someone’s movement that’s just normal movement, just restless or getting up and it’s not necessarily an emergency? We don’t want to install cameras, but I don’t know without a camera how you would know that.

The most common sentiment was that there is a balance to be struck between privacy and safety, though respondents indicated no process for achieving it.

Participants identified a tension between the needs and desires of beneficiaries and those of care providers:

There’s a consent factor there, and understanding if the person knows they’re being monitored, and if their representative is solely safety-driven and doesn’t include evaluation of dignity at risk. So there are a lot of factors. How informed is the person?

A state manager explained that they receive requests for cameras from family members to monitor people with Alzheimer’s disease and related dementias while away at work, which raises similar questions.

I know that that’s been a big trend in a lot of the different states. I know that it’s something that we probably need to start venturing in here to looking at what would that look like, what would the reimbursement look like? Under what circumstances would we allow something like that? I think we’ve got to be careful too that just because the family gets peace of mind, it’s like, well, we don’t want to invade that person’s right because we’re also struggling with that whole person-centeredness and having to take everyone’s perspective into account. You have to be very considerate of what that could look like. So I think there could be some ethical concerns and maybe some conflict.

Others expressed similar concerns about the use of GPS for beneficiaries with dementia:

Yes it’s great for family, but is it bad for a consumer who gets anxious?

These concerns were not linked to formalized processes for mitigating them. With few exceptions, individual support coordinators determined and safeguarded the balance between privacy and safety without technology training, additional supports, or consent form processes. Participants noted the need for research on participants’ experiences, ethical implications, and efficacy to help inform their decision making.

## Discussion

### Research and Policy Priorities

Service providers and policy makers recognize the need to monitor the safety of a growing population of older adults living alone in the community. The combination of looming federal cuts to Medicaid and a widespread shortage of attractive direct care jobs and workers have created a strong incentive to reduce reliance on personal aides [48].

In addition to these pressure points, the transition states are making to MLTSS is likely to increase the use of passive remote monitoring technologies. States have reported that they struggle with lack of data from MCOs to measure quality impact, particularly for duals [49]. Moreover, this research finds that critical feedback loops are lacking without mechanisms for tracking which beneficiaries are using which technologies in MLTSS and non-MLTSS states. Given the pace of innovation and onerous waiver application processes, states want service categories that do not restrict adoption of promising technologies, yet current categories do not accurately describe covered technologies. A move to categorize these by function (ie, scope of data collected; granularity; frequency) may help to solve both the flexibility problem and the information problem for overseers.

Ideally, those responsible for making policy decisions and for implementing policy through Medicaid programs would be in conversation with researchers on monitoring technologies; effective safeguards require knowledge of the nature of risks they are intended to mitigate. It is not unusual for research and research dissemination to lag behind policy and practice, but in this case, the conversation about the difficult trade-offs for Medicaid beneficiaries is lagging behind as well.

One place to start is the simple but profound acknowledgment that there is no such thing as technology [50]. Dutch anthropologist, Jeanette Pols reminds us that while there are many technologies, technology is just a concept. It is better to study and understand the implications of a specific technology than to place undirected hope in *technology* to categorically solve problems of resource restrictions in the face of demand growth. It is important, in other words, to distinguish an assistive technology like a brail embosser or a walker from a remote monitoring technology such as a movement sensor. The distinctions matter because their implications for actors and relationships differ. As participants pointed out, even within the category of passive remote monitoring, it is important to distinguish between GPS location tracking, sensors, and Web-camera monitoring. This study finds that this is not current practice. Participants prioritized 3 research aims that align with Pols’ encouragement to capture specificity: (1) identify what type of information beneficiaries want to be generated, whom they want it to be shared with, and under what circumstances, (2) understand how to support decision making for beneficiaries with cognitive impairment, and (3) evaluate the efficacy of various categories of remote monitoring technologies in relation to the costs.

This research should also inform the following policy priorities: (1) regulatory checks on risks and negative impact of passive remote monitoring; (2) training for support coordinators (care managers) and state staff on ethical implications across settings and uses; and (3) cross-state sharing of best practices and lessons learned. Regulatory checks require specific coding to inform states and CMS when a given technology is in use. This would enable tracking these technologies’ associations with positive or negative outcomes, which has the potential to greatly inform use in the absence of large randomized controlled trials. The implementation of such regulatory checks could also provide more intentional opportunities for beneficiaries and their families to request changes to their technology service according to their own experiences with it over time and as their conditions and situations change.

Across states, respondents reported uneven or nonexistent trainings for care coordinators and eagerness to learn from other states about emerging solutions to the difficulties of managing an ethically fraught intervention without a sound evidence base. Medicaid program managers are accustomed to difficult decisions forced by resource restrictions; however, the job of regulating the lesser of what may be considered two undesirable choices (being monitored remotely or move to a nursing home) may be especially complex. Put directly, a solution that is less bad than residence in a nursing home does not make it a sound ethical solution because beneficiaries may be made to accept undesirable circumstances [24]. In practice, for beneficiaries with decision-making capacity, ethical deployment of passive monitoring requires freely given informed consent and awareness that consent can be withdrawn [51]. One state was in the process of piloting a consent form, which included “privacy” as a checkbox; however, researchers are just beginning to learn what privacy means in relation to in-home monitoring and how to talk about it with older adults [31,52]. The known risks of passive remote monitoring, including loss of privacy, autonomy, control and human contact, require clear and careful articulation in relation to the nascent research. New efforts that are needed both to introduce training on ethical implications and to share best practices across states can be integrated, with opportunities for open discussion among practitioners about what they are encountering in the field.

### Conclusions

Medicaid waiver programs require flexibility to meet beneficiaries’ individual needs, and technology to support connectivity, well-being, and home care are important areas for growth in waiver programs. Nevertheless, when the economic logic of an intervention for “high-need, high-cost” individuals [53] is as powerful as it is in this case of Medicaid, we must be vigilant about what types of technologies are being integrated without an understanding of what constitutes appropriate use. Before investment in passive remote monitoring technology makes it difficult to reevaluate use, policy makers should heed gerontologists’ concerns regarding the parallels between monitoring and “processes of institutionalization,” cautioning that “careful consideration is necessary to ensure that programs, policies and technologies that are intended to contain costs by ‘protecting’ the health of older adults do not further disempower this already potentially marginalized group of individuals” [29]. The fact that struggling Medicaid programs are moving first into this uncharted territory adds weight to these concerns.

## Abbreviations

LTSS: long-term service and supports
MLTSS: managed long-term service and supports
PERS: personal emergency response systems
HCBS: home and community-based services
MCO: managed care organizations
CMS: Centers for Medicaid and Medicare Services
